# Bluetongue in the Mediterranean Basin: An Overview of Recent Hotspots and Advances in Vaccine Technologies

**DOI:** 10.3390/microorganisms14020437

**Published:** 2026-02-12

**Authors:** Ikram Joubair, Abdellatif Errabbani, Soukaina Daif, Jesus Zueco, Salim Bounou, Ouafaa Fassi Fihri, Ismaïl Moukadiri

**Affiliations:** 1 Engineering School in Biomedical & Biotechnology, Euromed Faculty of Pharmacy, Euro-Mediterranean University of Fez, Eco-Campus UEMF, Route de Meknes (RN6, Rond-Point Bensouda), Fez 30070, Morocco; i.joubair@ueuromed.org (I.J.); s.bounou@ueuromed.org (S.B.); 2 Unidad de Microbiología, Facultad de Farmacia, Universidad de Valencia, Avda. Vicente Andrés Estelles s/n, Burjassot, 46100 Valencia, Spain; 3 MPCAE Laboratory-Materials, Natural Substances, Environment and Modeling Team (MSNEM), Polydisciplinary Faculty of Taza (FPT), Sidi Mohamed Ben Abdellah University (USMBA) of Fez, Taza BP 1223, Morocco; 4 Pathology and Veterinary Public Health Department, Institut Agronomique et Vétérinaire HASSAN II, P.O. Box 6202, Rabat 10000, Morocco

**Keywords:** bluetongue virus (BTV), epidemiology, serotypes, Mediterranean basin, virus outbreaks, vaccination, next generation vaccine, DIVA

## Abstract

Bluetongue (BT) is a noncontagious, arthropod-borne viral disease of domestic and wild ruminants caused by bluetongue virus (BTV), an arbovirus of the *Orbivirus* genus within the *Sedoreoviridae* family. At least 36 serotypes have been identified globally; recurrent circulation of BTV-1, -4, and -8, along with the recent emergence of BTV-3 in northern Europe, underscores a persistent incursion risk for Mediterranean herds. Key drivers include climate-driven expansion of *Culicoides* vector niches, windborne dispersal, animal movements, and subclinical reservoirs in cattle and goats. As no specific treatment is currently available, control of bluetongue disease still relies largely on vaccination. Live-attenuated vaccines and inactivated vaccines have reduced incidence, but important limitations persist: risk of reversion and the possibility of reassortment for LAVs; requirement for multiple doses and limited cross-protection for inactivated products; and the absence of DIVA capability for both. As an alternative, next-generation platforms are under active evaluation. Subunit formulations, often VP2 combined with VP5 and/or NS1/NS2 virus-like particles (VLPs), and viral-vectored constructs demonstrate favorable safety, strong humoral and cellular responses, inherent or engineered DIVA compatibility, and potential for rapid updating against emergent serotypes. This review synthesizes recent bluetongue activity across the Mediterranean Basin and provides a critical assessment of both existing and emerging vaccine strategies, with a focus on recommending next-generation platforms that emphasize DIVA-compliant, multiserotype, and adaptable vaccination approaches, supported by integrated surveillance and vector control in the region.

## 1. Introduction

Ovine, bovine, and other ruminants are susceptible to bluetongue (BT), an arthropod-transmitted viral disease infecting both domestic and wild ruminants. It is a noncontagious infection caused by the bluetongue virus (BTV), a non-enveloped, double-stranded RNA (dsRNA) virus classified under the genus *Orbivirus* within the *Sedoreoviridae* family. The BTV genome is approximately 19 kbp in length and consists of ten segments. These segments encode seven structural (VP1–VP7) and five nonstructural (NS1, NS2, NS3/3A, NS4, and NS5) proteins [1,2].

To date, at least 36 serotypes of BTV have been identified worldwide [3,4]. The virus is transmitted between animals through competent *Culicoides* spp. vectors [5], linking regional epidemiological patterns directly to vector distribution, seasonal dynamics, and environmental change. Bluetongue was first detected in Africa before spreading to the Mediterranean Basin, where it was reported for the first time in Cyprus in 1943, and subsequently expanded to other regions. Over the decades, the Mediterranean region, defined in this review as countries and territories bordering the Mediterranean Sea, has emerged as a hotspot for repeated incursions and reemergence events involving multiple serotypes [6,7], underscoring the need for adaptive and forward-looking control strategies. While the primary focus of this study is the Mediterranean basin, references to certain non-Mediterranean countries may be included where they provide relevant epidemiological or vaccinology insights.

Although there is no curative treatment for bluetongue, vaccination has been successfully used to control outbreaks [6]. Vaccination against bluetongue started quite early with a monovalent vaccine based on a live attenuated virus, which was developed through repeated passages of the native virus in sheep and was used between 1907 and 1943 [6]. Thereafter, inactivated vaccines were introduced on the market in 1975 [8]. More recently, a recombinant vaccine based on the VP2 BTV outer capsid protein was developed. This vaccine is considered completely safe because of its recombinant nature, but, unfortunately, the VP2 subunit vaccine induces only low protective immunization and needs to be supplemented with adjuvants to increase the immunogenicity of the single protein [9]. Currently, advances in biotechnology and nanotechnology have led to the emergence of next-generation vaccines, such as nucleic acid-based vaccines and virus-like particle (VLP) vaccines, which have been developed and tested against hepatitis B, COVID-19, and several other diseases [10,11,12].

In light of these dynamics, this review aims to describe the recent epidemiological trends and situation of Bluetongue disease in the Mediterranean Basin and the prospects for next-generation vaccines that can alleviate this problem. We overview recent outbreaks and circulating serotypes in the region, review the evolution of vaccination strategies, from conventional approaches to novel platforms, emphasizing future vaccine prospects that could improve bluetongue control.

## 2. Historical Emergence and Global Spread of Bluetongue Virus

Bluetongue disease was reported for the first time over 100 years ago following the introduction of European sheep breeds into South Africa. This disease was initially described in this context by Spreull [13]. Early reports characterized the disease as a malarial catarrhal fever and termed it “bluetongue’’ by Henning et al. 1956, a term that was the result of the English translation of the Afrikaner term “Blaauwtong” by Spreull 1905. Subsequent studies demonstrated that the causative agent was a filterable virus, leading to the development of early live-attenuated vaccines derived from serial passage of native bluetongue virus strains in sheep [14,15]. For a long period, Bluetongue was largely confined to Africa. However, it has since spread to Europe, Asia, the Americas, and Oceania, with at least 36 BTV serotypes identified to date [16]. Bluetongue was identified for the first time in the Mediterranean basin in Cyprus in 1943, marking its regional emergence. The disease subsequently spread rapidly across the region and eventually reached more distant areas, including Canada, Australia, and the USA, ultimately dispersing worldwide [17].

## 3. Bluetongue Virus: Structure, Cell Entry, and Replication

### 3.1. Virion Architecture

BTV is an arthropod-borne *Orbivirus* belonging to the *Sedoreoviridae* family. Transmission occurs via hematophagous midges of the *Culicoides* genus [18]. BTV is a non-enveloped virus with an icosahedral structure. Its capsid is double-shelled and made up of seven structural proteins (VP1 to VP7).

The outer shell is composed of 60 VP2 trimers and 120 VP5 trimers [19], enclosing the inner shell or core, which consists of 120 monomers of VP3 and 260 trimers of the VP7 protein [20]. Within this core reside the 10 double-stranded RNA (dsRNA) segments that constitute the BTV genome. VP2 is the most exposed surface protein, featuring a spike-like shape and a molecular mass of ~110 kDa. It determines the viral serotype, mediates hemagglutination, and plays a central role in host cell attachment [21,22]. VP5, the second most exposed protein, has a roughly spherical structure with a size of ~60 kDa. It is essential for viral penetration, enabling the release of the transcriptionally active core into the host cell cytoplasm [20].

### 3.2. Virus Entry, Replication Cycle, and Release

**Figure 1:**
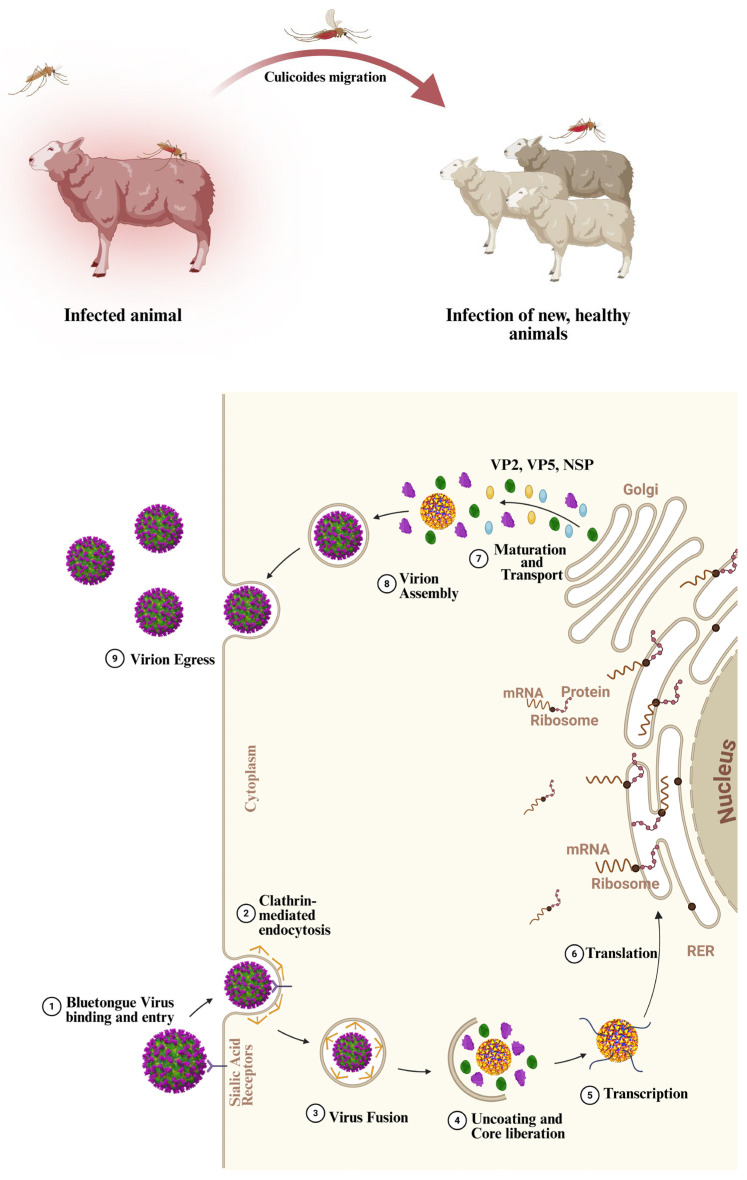
BTV transmission, cell entry, and life cycle. Schematic representation of the BTV life cycle, including viral attachment and entry via clathrin-mediated endocytosis (CME), intracellular synthesis of structural and nonstructural viral proteins (VPs/NSPs), including translation on cytosolic ribosomes and at the rough endoplasmic reticulum (RER), and assembly and release of progeny virions. Created in BioRender. Joubair, I. https://BioRender.com/z666674, https://BioRender.com/55lku1w (accessed on 9 April 2025).

BTV outer capsid layer is relatively unstable, and this instability plays a crucial role in BTV cell entry [20]. BTV is internalized through clathrin-mediated endocytosis (CME). Within the endosomal compartment, the acidic pH triggers the dissociation and degradation of the VP2 protein, thereby exposing VP5. The fusogenic activity of VP5 subsequently destabilizes the endosomal membrane, allowing the transcriptionally active BTV core to escape into the host cell cytoplasm [23]. Once the transcriptionally active core is released into the cytoplasm, the ten dsRNA genomic segments are transcribed, generating ten single-stranded RNAs (ssRNAs) that serve as messenger RNAs (mRNAs). Host cell ribosomes translate these mRNAs, leading to the synthesis of seven structural proteins (SPs) and five nonstructural proteins (NSPs) [19]. Among these proteins, NSP1 plays a pivotal role in regulating gene expression by promoting the translation of ssRNA transcripts, leading to the synthesis of other proteins, with a corresponding increase in the BTV protein concentration in the host cell [24]. When all BTV proteins are synthesized and ready to assemble, nonstructural protein 2 (NSP2) begins to build the BTV structure, starting with the establishment of globular structures called viral inclusion bodies, which recruit the remaining components that make up the BTV core. Because of its ssRNA-binding capacity, NSP2 subsequently recruits BTV ssRNAs in a process in which VP6 also participates, and the dsRNA genomic segments are synthesized by VP1 (RNA polymerase), completing the BTV transcriptional subcore [25]. At this stage, the VP7 trimers assemble onto the BTV subcores by interacting with the VP3 proteins. Finally, following the acquisition of the outer capsid proteins VP2 and VP5, fully assembled BTV particles exit the host cell via vesicular trafficking pathways and propagate the infection to new cells [23,26] (Figure 1).

## 4. BTV Serotypes

Among BTV proteins, VP2, the outer capsid protein, is the main determinant of BTV serotypes. BTV comprises at least 36 serotypes (BTV-1 to BTV-36), which differ mainly in the VP2 capsid protein [16,27]. A comparison of BTV serotypes based on the VP2 nucleotide sequence revealed considerable variation. For example, the degree of similarity between BTV-8 and BTV-18 based on the VP2 nucleotide sequence is 29%, whereas the similarity between BTV-16 and BTV-22 reaches 59%. Comparisons of VP2 amino acid sequences show a similar trend: the percentage of variation between VP2 of BTV-4 and BTV-20 is 22.4%, while the variation between VP2 of BTV-6 and BTV-22 reaches 73% [28]. These percentages confirm that the VP2 protein of one serotype may differ significantly from that of another [28]. This contrasts with the VP5, VP3, and VP7 proteins, which are relatively conserved across all BTV serotypes. For example, the percentage of identity between the VP5-BTV-13 and VP5-BTV-10 amino acid sequences isolated from the USA is 82%, and the percentage of variation in the VP3 protein of BTV-15 and the same protein of other BTV serotypes is only 4% in the amino acid sequence and 20% in the nucleotide sequence, confirming the conservation of the VP3 and VP5 capsid proteins among BTV serotypes.

VP2 is therefore the most variable BTV protein and the principal determinant of serotype diversity [29]. To further complement the data presented above, the nucleotide sequences of the VP2, VP3, VP5, and VP7 proteins from serotypes 1, 4, and 8 were analyzed using the BLAST tool (https://blast.ncbi.nlm.nih.gov/Blast.cgi?PROGRAM=blastn&PAGE_TYPE=BlastSearch&LINK_LOC=blasthome, last access date on: 22 December 2025) provided by the National Center for Biotechnology Information (NCBI). The results of these comparisons are summarized in Table 1. Comparison of the VP2 nucleotide sequences showed that serotypes 1 and 4 share 56.3% identity and 56.6% similarity, whereas serotypes 1 and 8 exhibit 59.1% identity and 59.5% similarity. Notably, the comparison between serotypes 4 and 8 revealed a substantially higher nucleotide identity (73.91%), indicating a closer genetic relationship at the VP2 level.

The VP5 nucleotide sequences of serotypes 1 and 4 exhibited 68.6% identity and 69.2% similarity, whereas serotypes 1 and 8 showed 72.7% identity and 73.4% similarity. Similarly to VP2, VP5 exhibited a higher level of nucleotide conservation between serotypes 4 and 8 (70.43% identity); however, this conservation was less pronounced than that observed for VP2. In contrast, comparisons of the VP3 and VP7 nucleotide sequences among serotypes 1, 4, and 8 consistently show high identity and similarity values exceeding 90% in all pairwise comparisons, with VP7 identities reaching up to 96.71% and VP3 identities up to 96.18%. These results confirm that VP2 is the most variable viral protein and the primary determinant of serotype, followed by VP5, which shows moderate inter-serotype variability. At the same time, VP3 and VP7 are highly conserved proteins that contribute minimally to serotype specificity (Table 1). This marked variability has real consequences for vaccine design. Protective immunity against BTV is predominantly serotype-specific and largely driven by neutralizing antibodies targeting VP2, meaning that protection against one serotype usually offers little or no cross-protection against others. As a result, effective bluetongue vaccines need to either include multiple serotypes in multivalent formulations or target more conserved viral antigens, such as VP7 or VP3, to potentially induce broader cross-protective immune responses [30].

## 5. BTV Epidemiology and Associated Outbreaks Affecting the Mediterranean Basin: From 2004 to Date

The first Bluetongue outbreak outside Africa occurred in Cyprus in 1943, resulting in approximately 2500 sheep deaths, followed by an outbreak in Turkey in 1945, which caused over 2700 sheep deaths, as reported by the Ministry of Agriculture [6]. In 1951, a bluetongue serotype designated BTV-4 was detected in Israel [29,31]. Several decades later, between 2003 and 2005, the same serotype was identified in several Mediterranean countries, including Portugal, Spain, and the island of Corsica (France), having spread from North Africa.

In August 2006, the first BTV-8 infections were detected near Maastricht, the Netherlands. Soon after, additional cases were identified in Belgium, Germany, France, and Luxembourg [32]. Germany reported 885 outbreaks, Belgium confirmed 675 cases, and France and Luxembourg were also affected. In Germany, BTV-8 caused estimated economic losses of approximately 157–203 million euros [33]. Comparative analyses later showed that BTV-8 shared high genetic similarity with BTV strains previously isolated in sub-Saharan Africa, highlighting the capacity of Mediterranean and African BTV lineages to disseminate beyond their initial geographic range [17].

Morocco, a Mediterranean country, remained largely free of Bluetongue for an extended period, from the first emergence of the disease until 2004. Prior to this, BT was considered an exotic disease, except for a few cases of BTV-10 detected in 1956, traced to Portugal [34]. It was not until 2004 that a series of outbreaks were reported, with the BTV-4 serotype responsible for these events following its spread across the Mediterranean basin [35]. Approximately two years later, the BTV-1 serotype was introduced into Morocco from Algeria. This serotype subsequently spread nationwide, resulting in 505 outbreaks [36]. Nevertheless, after a few months without reported cases, BTV-1 reemerged in 2006, causing 1076 outbreaks and 2180 deaths between May and November. Overall, all Bluetongue outbreaks in Morocco have been attributed to three major serotypes: BTV-1, BTV-4, and BTV-8 [36].

In Europe, BTV-16 was reported and isolated in Corsica in 2004. Comparative studies have shown that this BTV-16 serotype is closely related to the BTV-16 strain used in the Onderstepoort vaccine, produced in South Africa, raising questions regarding vaccine-derived virus circulation [37]. In Portugal, the first identification of BTV was reported in 1956, and successive outbreaks continued until 1960. From 1960 to 2004, for more than 40 years, Portugal remained free of bluetongue. However, in October 2004, BT was reintroduced, leading to a series of outbreaks caused by the BTV-4 serotype. Several strains were subsequently collected and analyzed. Genomic analyses revealed strong links with BTV-4 strains circulating in Sardinia and Corsica reported in 2003. In July 2005, a new outbreak caused by BTV-2 was reported, with isolates showing high genetic similarity with BTV-2 strains previously identified in Italy and a South African BTV-2 vaccine strain. In 2006, additional outbreaks of BTV-4 were detected in central Portugal [38].

By 2007, the BTV-1 strain first reported in Morocco and Algeria had spread to Sardinia (Italy) and subsequently to other Mediterranean countries, including Portugal, Spain, and France [37]. Between 2004 and 2010, the Mediterranean basin experienced successive and overlapping BTV outbreaks involving multiple BTV serotypes. From Morocco, BTV-4 crossed into Spain and spread there during 2005–2006. In 2007, a new BTV serotype was isolated in southern Spain and identified as BTV-1. Nevertheless, by the end of the decade, Spain experienced yet another outbreak caused by BTV-4 [34]. During the same period, BTV-6 was reported in the Netherlands and Germany, BTV-25 was detected in Switzerland, and BTV-11 was identified in Belgium. This BTV-11 outbreak and the aforementioned BTV-6 outbreak were suspected to be related to specific vaccination campaigns [17].

In 2012, Sardinia (Italy) experienced outbreaks caused by both BTV-4 and BTV-1. The BTV-4 serotype identified was confirmed to be closely related to BTV-4 isolates previously reported in Tunisia in 2007 and 2009 [37]. Less than a year later, in October 2013, cases of Bluetongue were reported on the island of Corsica (France) and identified as BTV-1. The outbreak rapidly spread across the island, with an estimated morbidity rate of 80% and a mortality rate of approximately 11%. Molecular analyses suggested that this serotype was introduced into France from Sardinia [39]. After approximately six months, a new BTV serotype, identified as BTV-4, spread through several Mediterranean countries in 2014, starting in Greece in May 2014 and then expanding to the rest of Europe. By the end of the year, the majority of Mediterranean countries had been affected, especially European countries, including Croatia [40]. The morbidity and mortality rates associated with this BTV-4 outbreak were estimated at 15.3% and 4.5%, respectively. Genome sequence analyses of circulating BTV-4 strains revealed close genetic relationships with BTV-1, BTV-2, and BTV-4 isolates previously circulating in North Africa and the Western Mediterranean [40,41].

In 2015, BTV-1 caused some 600 outbreaks in Morocco, with a morbidity rate of 2.45% and a fatality rate of 0.67% [36]. During the same year, BTV-8 re-emerged in France, with genomic analysis showing high similarity between the circulating French strain and BTV-8 strains previously reported in Mediterranean countries between 2007 and 2009 [42]. Nevertheless, in November 2016, new clinical cases of BT were reported in France, and the responsible serotype was identified as BTV-4, which was likely introduced from Italy [43]. The same BTV-4 serotype invaded France from Corsica in June 2017, reaching the mainland in November of the same year and causing multiple outbreaks in sheep and cattle [44]. In 2017, severe BT cases caused by the BTV-6 serotype, characterized by classical clinical signs and deaths, were reported on sheep and cattle farms in Israel, with outbreaks spreading rapidly across the country. Genetic and phylogenetic analyses based on genomic segments of the BTV-6 serotype sequenced in Israel have revealed a relationship between this serotype and other Mediterranean BTV serotypes. For example, comparison of segment 2 of this serotype with the same segment of the African BTV-6 reference strain showed 93.88% identity, while analyses of additional genomic segments demonstrated high levels of homology (97% to 99.47% identity) with BTV-3, BTV-4, and BTV-9 strains circulating in the Mediterranean basin [31]. One year later, in 2018, BTV-8, responsible for the French outbreaks in 2015, spread to Switzerland and Germany. At the same time, BTV-1 spread from Spain to Portugal [37,45]. In 2019, BTV-8 was detected in Belgium, most likely following a northward spread from Mediterranean regions.

**Figure 2:**
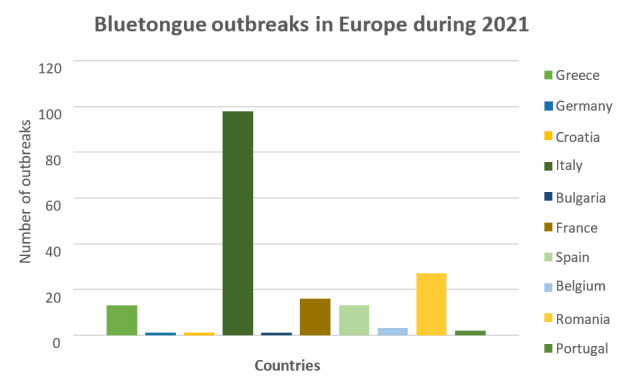
Reported bluetongue disease outbreaks in selected European and Mediterranean basin countries during 2021. The figure summarizes the number of officially reported outbreaks in Greece, Germany, France, Spain, Belgium, Portugal, Croatia, Bulgaria, Italy, and Romania based on national and international animal health surveillance data for the year 2021.

A recent study on a BTV-4 outbreak in Tunisia estimated the total mean cost of the outbreak at ~1935 million Tunisian Dinars (~€561 million) across surveyed farms, with significant losses due to decreased milk yield, mortality, veterinary treatment, and weight loss [46]. In 2021 alone, the bluetongue virus caused over 200 outbreaks in the Mediterranean basin within just six months. The European countries most affected that year were Italy (98 outbreaks), Romania (27), France (16), Spain and Greece (13 each), Belgium (3), Portugal (2), and Bulgaria, Croatia, and Germany, each reporting one outbreak [37,45] (Figure 2). Finally, in September 2023, BTV-3 was identified in the Netherlands, following reports of bluetongue-like clinical signs in sheep (Netherlands Food and Consumer Product Safety Authority), with subsequent confirmation in cattle. Although outside the Mediterranean Basin, this emergence is of concern due to the demonstrated capacity of BTV to spread and re-enter the Mediterranean regions [47].

Taken together, these successive outbreaks highlight the persistent circulation and expanding epidemiological complexity of bluetongue virus affecting the Mediterranean Basin. This situation is further reinforced by the presence of multiple domestic and wild reservoir hosts, including sheep, goats, cattle, and camels, that sustain viral transmission [37]. Bluetongue incursions have placed a substantial and recurring economic burden on the livestock sector. At a global level, BT is estimated to cost the livestock industry approximately US$3 billion annually in production losses and disease control efforts [48]. In Mediterranean countries, these losses are primarily driven by direct on-farm impacts, including high mortality and culling in sheep, reproductive failures, reduced growth rates, and marked declines in milk production [6,46].

## 6. Bluetongue Vaccines: Evolving Platforms, and Policy Considerations

Currently, there is no curative treatment for animals infected with BTV [8]. Therefore, preventive vaccination, integrated with animal movement control and vector management, remains the most effective and sustainable strategy to limit the spread of the disease. Vaccination programs against BT significantly reduce economic losses by lowering mortality, morbidity, fetal abortions, and other clinical manifestations, including reduced milk and meat production [6].

Effective vaccination depends on the capacity to engage the same innate and adaptive immune mechanisms activated during natural infection. A coordinated humoral and cell-mediated immune response is triggered by a bluetongue virus infection [49].

Following viral entry, antigen-presenting cells internalize the virus and initiate innate immune signaling, prominently through the induction of type I interferons (IFN-α/β) [50]. This early response supports the subsequent activation of adaptive immunity, including CD4^+^ helper and CD8^+^ cytotoxic T lymphocytes, as well as the production of virus-specific antibodies [50]. Neutralizing antibodies are primarily directed against the outer capsid proteins VP2 and VP5 and are responsible for serotype-specific protection. However, the high genetic and antigenic variability of VP2 limits cross-serotype neutralization. In parallel, cellular immune responses, particularly those mediated by NS1-specific CD8^+^ T cells, play a critical role in viral clearance and provide a degree of cross-serotype immunity [51,52]. CD4^+^ T cells further support B-cell maturation and the establishment of long-term immunological memory. Together, these immune mechanisms underpin current strategies for the rational development of effective BTV vaccines [51,52].

Adjuvants play a pivotal role in enhancing the magnitude, quality, and durability of immune responses induced by BTV vaccines, particularly for inactivated and subunit formulations. Oil-based adjuvants, aluminum salts, saponins, and modern adjuvant systems improve antigen uptake and presentation, promote neutralizing antibody production, and in some formulations enhance T-cell-mediated immunity. Appropriate adjuvant selection is therefore critical for optimizing balanced humoral and cellular immune responses against Bluetongue virus [53,54].

Bluetongue vaccination strategies rely on both inactivated and live attenuated vaccines, with a wide range of commercial vaccines available (Table 2). These products are generally effective, although they differ in their risk–benefit profiles [53,55].

### 6.1. Live Attenuated Vaccines

Live attenuated vaccines (LAVs) or modified live vaccines (MLVs) are created by multiple successive passes of the virus through specific or nonspecific host cells, such as embryonated chicken eggs (ECEs) or baby hamster kidney cells (BHKs). The process leads to reduced virulence of the virus through the natural accumulation of mutations, which ultimately hinder the ability of the virus to replicate efficiently in specific host cells [11]. This method was first introduced by Theiler [56], who was honored with the Nobel prize in physiology and medicine for the creation of the yellow fever vaccine using this method in 1951 [57]. Early bluetongue immunization efforts date to 1906, when the first live attenuated vaccine was developed against the BTV-4 serotype by serial passaging of the virus in sheep. This vaccine has been used in South Africa and worldwide for over four decades with good results, demonstrating efficacy with an estimated 11% reduction in the mortality rate [11]. Thereafter, several LAVs targeting BTV-14 and other serotypes have been commercialized in South Africa. All of these vaccines were developed by attenuating the native virus through multiple serial passages in embryonated chicken eggs (ECEs), in some cases exceeding 100 passages. These vaccines protect vaccinated animals against specific serotypes. However, they do not confer cross-protection against unrelated serotypes [11]. Since the introduction of the first Bluetongue vaccine in 1906 to date, many LAVs have been produced against different BTV serotypes. These include BTV-8 in 1937; BTV-12 in 1941; BTV-9 in 1942; BTV-11 in 1944; BTV-5 in 1953; BTV-7 in 1955; BTV-1, BTV-2 and BTV-6 in 1958; BTV-13 and BTV-14 in 1959; BTV-19 in 1976, developed in the Republic of South Africa, and; BTV-3 in 1944; and BTV-10 in 1956, which were developed in Cyprus and Portugal, respectively, using BHK cells for virus passages [58]. It has been common for countries to develop vaccines in response to outbreaks caused by specific BTV serotypes. For example, the state of California in the USA developed LAVs targeting serotypes 10, 11, and 17; China developed vaccines against serotypes 1 and 16; and Turkey and Morocco developed vaccines against the BTV-4 serotype [58,59] (Table 2).

**Table 2:** Vaccines that have been developed against bluetongue.

Vaccine Platform	Examples (EU, Mediterranean, or Experimental)	Serotype (S) Targeted	Administration Route	Safety	Immunogenicity/ Efficacy	Booster Requirement	DIVA Capability	Costs	References
Live attenuated	BTVAC Bivalent BTV-1, BTV-4 (Biopharma)	BTV-1, BTV-3, BTV-4, BTV-10, BTV-11, BTV-16 and BTV-17	Subcutaneous	Low	Very good	Yes	No	+	[11,12,58,60]
Inactivated Vaccines	General formulations	Monovalent (BTV-2, BTV-4, BTV-8, BTV-11, BTV-16, BTV-17, BTV-18), Bivalent (BTV-1 and -23), (BTV-4 and -2), (BTV-1 and -4), (BTV-4 and -16), and Pentavalent (BTV-1, 2, 15, 18 and 23)	Subcutaneous/Intramuscular	Very High	Good	Yes	No	++	[6,11,12,53,55,58,59,61,62,63]
	BLUEVAC	BTV-1, -4, -8	Subcutaneous (Sheep and Cattle)	High	Good	Yes	No	++	[64]
	Bovilis BTV8	BTV-8	Subcutaneous (Sheep and Cattle)	High	Good	Sheep: 1 dose primary + revaccination at 12 months; Cattle: 2 doses primary (3-week interval) + revaccination at 12 months	No	++	[65]
	Syvazul BTV	BTV-1, -4, -8	Subcutaneous (Sheep) Intramuscular (Cattle)	High	Good	In Sheep: 1 dose primary + revaccination at 12 months Cattle: 2 doses primary (3-week interval) + revaccination at 12 months	No	++	[66]
	Bultavo-3	BTV-3	Subcutaneous (Sheep) Intramuscular (Cattle)	High	Good	Sheep: 1 dose primary; Cattle: 2 doses primary (3-week interval) revaccination not established	No	++	[67]
Subunit vaccines	Experimental	BTV-10 (VP2); BTV-4(VP2 and VP5), BTV-8 (VP2, NS1 and NS2), BTV-16 (VP2, VP5, VP3, VP7 and NS2), BTV-8 (VP2, VP5, NS1, NS2 and NS3)	Typically intramuscular or subcutaneous (parenteral) with adjuvant	Very high	Weak	Yes	Yes	++	[11,12,38,68,69,70,71]
VLPs vaccines	Experimental	BTV-1, BTV-2, BTV-8, BTV-10, BTV-13, BTV-17	Typically intramuscular or subcutaneous (parenteral), often multi-dose	Very high	Good	Yes	Yes	+++	[11,12,71,72]

Safety: Low, High, and Very High indicate increasing levels of vaccine safety. Cost: Relative production and implementation cost. + = low cost; ++ = moderate cost; +++ = high cost. Immunogenicity/Efficacy: Qualitative evaluation based on experimental and field data. Weak indicates limited or inconsistent immune protection; Good indicates reliable protective immune responses; Very Good indicates strong and consistent protection.

Live attenuated vaccines (LAVs) have been widely used across the globe to control bluetongue outbreaks. In the Mediterranean basin, which is one of the regions most affected by Bluetongue, LAVs have been frequently used as a control strategy. For example, the first BT outbreak in Turkey, caused by BTV-4, was reported in 1977, persisted until 1988, and was finally controlled through the use of an attenuated vaccine. Similarly, in the same area, BTV-9 caused the first BT outbreak in Bulgaria in 1999, affecting over 80 villages. The situation prompted the Bulgarian government to implement animal movement restrictions, vector control measures, and vaccination campaigns in 1999–2000 using South African LAV pentavalent vaccines against BTV serotypes 3, 8, 9, 10, and 11. These combined efforts ultimately succeeded in containing the outbreak [61].

In Morocco, the rapid application of vaccination with a polyvalent vaccine successfully stopped the spread of the disease, and from 1956 to 2004, this region was free from BT, except for some asymptomatic cases that tested positive during the surveys. However, in 2004, new BT outbreaks were reported along the northwestern Atlantic coast. The causative agent was a BTV-4 serotype closely related to European isolates, suggesting the European origin of the strain. Since the 2004 outbreak, various LAVs have been used in Morocco to control BT. From 2004 to 2008, a monovalent vaccine locally produced against BTV-4 was used for vaccination. Since 2008, vaccination campaigns have incorporated a new locally produced bivalent vaccine targeting both BTV-1 and BTV-4 [36].

LAVs are generally efficacious and induce robust cellular and humoral immune responses even at low doses. However, LAVs have some disadvantages related to animal and environmental safety, such as the transmission of LAV strains by *Culicoides* midges and the emergence of new virulent strains due to reversion or reassortment with the circulating strains. In addition, fetal abortions and a reduction in milk production have been reported as a result of vaccination with LAVs. Finally, LAVs do not allow the differentiation of naturally infected animals from vaccinated animals (DIVA), which is a serious drawback for the safe movement of animals without risk of BT disease expansion [11,73,74].

### 6.2. Inactivated Vaccines

The first description of an inactivated virus vaccine against Bluetongue was in 1975. The preparation of this type of vaccine consists of treating the native virus with chemical compounds, such as hydroxylamine, acetylethylenimine, formaldehyde, or beta-propiolactone, or alternatively, through physical methods, including ultraviolet (UV) radiation or heat treatment [73]. Since 1975, several inactivated Bluetongue vaccines have been developed and commercialized. In the European Union, for example, the first inactivated BT vaccine produced and used was a monovalent vaccine against the BTV-2 serotype. This was followed by a vaccine against BTV-4 and subsequently a bivalently inactivated vaccine targeting both BTV-2 and BTV-4. A more recent formulation included a bivalent vaccine against BTV-8, incorporating aluminum hydroxide and saponin as adjuvants.

In India, one of the countries significantly affected by Bluetongue, several BTV strains have been used to develop inactivated Bluetongue vaccines [6]. This includes a monovalent vaccine against BTV-2, another targeting BTV-18, bivalent vaccines against BTV-1 and BTV-23, and, finally, a pentavalent formulation including BTV-1, 2, 15, 18, and 23 [11,73,75]. More recently, in 2021, a new bivalent inactivated vaccine targeting serotypes BTV-1 and -4 was developed in Morocco. However, it remains experimental and is not yet commercially available [59].

Inactivated bluetongue vaccines have also been used worldwide for vaccination. This type of vaccine was commercialized for the first time in Europe in 2005. Vaccination of animals started with a vaccine against BTV-2 and then with monovalent and multivalent vaccines against BTV-1, -2, -4, -8, and -9 [14]. Across the European Union, millions of cattle, sheep, goats, and other susceptible animals have been vaccinated according to national policies. For example, in Italy, all susceptible animals can be vaccinated; in Spain, only sheep and cattle can be vaccinated, whereas in France, only sheep can be vaccinated with inactivated vaccines [58].

As with live attenuated vaccines, inactivated vaccines have been widely used to prevent the development of clinical signs of Bluetongue disease and to reduce viremia in ruminants, thereby contributing to a decrease in virus transmission and limiting the associated economic losses [75]. An inactivated BTV-8 vaccine was deployed in Europe in 2006 to contain the spread of the BTV-8 serotype, which played a key role in controlling the outbreak and substantially reducing economic losses [33,55]. Compared with LAVs, inactivated vaccines are safer in that they do not have the potential problems of virus reversion or the reassortment of new virulent vaccine strains when inactivation is properly performed [8,11]. On the other hand, inactivated vaccines have several limitations. First, as a consequence of the requirement for complete inactivation to ensure the absence of the residual infectious virus, they are more expensive to develop and produce. Inactivation also drastically decreases immunogenicity, requiring multiple doses with high concentrations to ensure protective immunity. Finally, as is the case with LAVs, there are no valid strategies for distinguishing infected animals from vaccinated animals (DIVA) [14,76].

### 6.3. Disabled Infectious Single Animal (DISA) and Single Cycle (DISC) Strategies

Genetically modified live vaccines, including Disabled Infectious Single Cycle (DISC), and Disabled Infectious Single Animal (DISA) platforms, are emerging as promising next-generation or controlling orbivirus infections such as bluetongue [12]. Both approaches rely on targeted gene deletions that limit viral replication and block transmission, which reduces the major safety concerns of conventional live attenuated vaccines [12,77].

DISC viruses are engineered by deleting an essential viral replication gene (for bluetongue virus, often the VP6 helicase), such that the virus can infect cells and express all viral antigens but cannot produce progeny [78]. In practice, a DISC virus enters a host cell and goes through one round of replication, expressing its full antigenic repertoire, but this abortive infection yields no infectious progeny. This single-cycle infection is enough to stimulate a robust immune response while preventing any further spread of the virus [12]. In contrast, DISA vaccines retain the capacity for repeated replication cycles within the vaccinated host. However, they are genetically engineered to be non-transmissible by targeting viral determinants involved in egress and vector competence.

In the BTV context, both platforms have demonstrated high levels of protective efficacy in ruminants [78]. DISC-based BTV vaccines are genetically modified to lack expression of the essential helicase VP6. Experimental studies in sheep and cattle have shown that both monovalent and multivalent DISC vaccines confer robust protection against clinical disease and viremia, while maintaining an excellent safety profile with no evidence of sustained viremia or transmission [78,79]. Likewise, DISA vaccines carrying NS3/NS3a deletions have demonstrated complete clinical protection in both sheep and cattle, with no detectable viremia and no transmission risk [61,80]. A prototype DISA8 vaccine (BTV8 outer proteins with a 72-aa NS3 deletion) in cattle was reported to be well tolerated and to induce durable, serotype-specific protection, while maintaining DIVA compatibility through the absence of NS3-directed antibody responses to distinguish wild-type infection from vaccination [81].

Collectively, DISC and DISA platforms meet key requirements for modern veterinary vaccines [82]. They overcome several shortcomings of traditional bluetongue vaccines, particularly the safety risks linked to live-attenuated strains and the lack of DIVA capability, making them promising options for inclusion in bluetongue control programs [12,61,83].

### 6.4. Subunit Vaccines

As we have already discussed, despite their efficacy and immunogenicity, both inactivated and live attenuated vaccines present certain limitations. For LAVs, the main concerns are linked to the risk of virus reversion and the possibility of reassortment with circulating strains, which may generate new virulent variants. In addition, neither LAVs nor inactivated vaccines allow the application of the DIVA strategy, preventing the differentiation of infected from vaccinated animals. In this context, recombinant subunit vaccines may offer a solution to both issues. Because these vaccines contain only selected viral proteins rather than the whole virus, they eliminate the risk of reversion or reassortment, and they are fully compatible with DIVA strategies [11].

Purified protein-based BT vaccines have been created either by combining various BTV proteins into a single vaccine preparation or by employing single proteins alone. More than 40 years ago, Huismans and colleagues [12,84] purified the BTV VP2 capsid protein by chemical extraction and later demonstrated that it induced good protective immunity against BT in vaccinated sheep due to the neutralizing characteristics of anti-VP2 antibodies [84]. The main limitation, as with all purified viral proteins, was the difficulty of producing VP2 in quantities sufficient for large-scale vaccine development. However, the revolution in reverse genetics and biotechnology has made cloning and expression of the VP2 gene and other BTV coding genes routine and less expensive [85,86].

Various subunit vaccines based on single BTV proteins, such as VP2, or on combinations of different proteins have been developed for treating BT disease. As previously noted, the earliest approach relied on the chemical purification of VP2 [68,84]. Several subsequent studies have demonstrated that combining VP2 with other BTV proteins can significantly reduce the antigen dose required to induce effective protective immunity [84]. For example, in vaccination-challenge studies conducted in sheep, VP2 alone requires a dose of 100 µg to induce protective immunity, whereas the addition of just 20 µg of VP5 reduces the required VP2 dose to 50 µg [87]. Building on this principle, several vaccines have been developed by combining VP2 with additional BTV proteins. One such example is a subunit vaccine against BTV-4 composed of VP2 and VP5, produced using a bacterial expression system. This vaccine was found to be immunogenic, inducing protective humoral and cellular immune responses in mice and offering the added benefit of being DIVA-compatible [69].

The BTV-8 outbreak that affected European countries in 2006 was characterized by a high incidence of clinical symptoms in cattle and an unusual teratogenic effect, which compromised traditional control methods that focused only on small ruminants. This has pushed researchers to develop vaccines capable of eliciting immunity in cattle. An experimental subunit vaccine was developed based on the VP2 structural protein of BTV-8 and the nonstructural proteins NS1 and NS2 of BTV-2. In cattle, this vaccine elicited a strong neutralizing antibody response against all three proteins [38]. Compared with commercial inactivated vaccines, the VP2, NS1, and NS2 subunit vaccines induced superior immunogenicity. Additionally, anti-VP7 antibody detection enables DIVA compliance, a feature that is not achievable with either inactivated or attenuated vaccine approaches [38]. Building on prior research, the same group developed an additional vaccine candidate combining the structural proteins VP2 and VP5 from BTV-8 with the nonstructural proteins NS1, NS2, and NS3 from BTV-2, formulated with an immunostimulant complex (ISCOM)-matrix adjuvant. This vaccine elicited robust cellular and humoral immune responses that were comparable or superior to those produced by a conventional commercial inactivated vaccine, remained fully DIVA compliant, and conferred protection against BTV-8 challenge in calves [70]. A similar strategy of combining structural and nonstructural proteins was used in China to create a subunit vaccine against the serotype BTV-16. In this case, the VP2, VP3, VP5 and VP7 structural proteins were combined with the nonstructural protein NS2 to induce protective immunity against the targeted serotype in sheep [68]. Different expression systems, including bacteria [69], baculovirus expression systems, and even plants [64,65], have been successfully used to express recombinant BTV proteins. This is the case for an experimental subunit vaccine based on the VP2 protein expressed *in Nicotiana benthamiana* via transient *Agrobacterium*-mediated expression. VP2 was accumulated in the cell plant cytoplasm and, after purification, it showed good results by inducing protective immune responses in mice without requiring the use of adjuvants [9,88]. All subunit vaccines include the crucial VP2 protein, either alone or in combination with other proteins such as VP5, VP3, VP7, NS1, and NS2. In contrast, subunit vaccines based solely on VP3 and VP7 (CLPs—capsid-like particles) are less immunogenic, making VP2 the critical component of all potential subunit vaccines [72].

### 6.5. Virus-like Particles: A Promising Approach

As an alternative to traditional vaccines based on cell culture viruses, virus-like particles (VLPs) represent a promising vaccination strategy against emerging viruses. VLPs are hollow, self-assembled particles that closely mimic the conformation of the native virus and contain the surface proteins involved in host cell entry. However, because they lack the viral genome, VLPs are unable to support gene expression, viral replication, or the formation of new virions. The structure and characteristics of VLPs closely resemble those of native infective BTV particles, and, potentially, VLPs may induce a stronger immune response and better protection than subunit vaccines. Their small size (approximately 20–100 nm) enables efficient entry into lymphatic vessels and lymph nodes, facilitating uptake by various types of antigen-presenting cells (APCs) [58,89]. VLP-based vaccines have long been used against both human and animal diseases. Notable examples include the hepatitis B virus-like particle vaccine introduced in 1986 and produced in yeast cells; the human papillomavirus VLP vaccine in 2006, which was produced both in yeast and insect cells; and the hepatitis E virus vaccine in 2011, which was produced in *Escherichia coli* cells [90]. Structurally, like viruses, VLPs can be categorized into enveloped and non-enveloped VLPs. Enveloped VLPs (eVLPs) are nanoparticles similar to enveloped viruses but lack the viral genome, acquiring their envelope from the cytoplasmic membrane of the host cell during expression and assembly. Nevertheless, designing, developing, and storing eVLPs is challenging, as their stability is limited. The fragility of the envelope makes these particles highly sensitive to environmental conditions. Indeed, temperature fluctuations and purification protocols can compromise VLP integrity, thereby reducing their immunogenicity [91,92].

The second kind of VLP is non-enveloped VLPs (neVLPs), which are protein-based nanoparticles that lack the lipid envelope derived from the host cell plasma membrane. NeVLPs represent the most effective form of assembled capsid proteins. They can serve as vaccines against a range of animal and human diseases, owing to their ease of development and purification [93,94].

Several enveloped and non-enveloped VLPs have been developed and tested as vaccines against various diseases. VLPs targeting human papillomaviruses (types 6, 11, 16, 18, 31, 33, 45, 52, and 58) were produced using a yeast expression system. In contrast, VLPs against hepatitis B were generated in Eukaryotic cells (Chinese hamster ovary -CHO- cells) and subsequently tested. The VLP approach can also be adapted to produce vaccines against non-viral pathogens, as illustrated by Mosquirix^TM^, a WHO-endorsed vaccine against malaria based on VLPs that contain both *Plasmodium falciparum* and hepatitis B antigens expressed in a yeast system. Other examples include VLP vaccines for seasonal influenza via the *Nicotiana benthamiana* plant expression system, Ebola virus and SARS-CoV-2 expressed in insect cells, hepatitis E virus in *Escherichia coli*, and HIV expressed in yeast. These vaccines are at different stages of development and clinical evaluation [91].

VLPs can display high-density B-cell epitopes for specific antibody production, as well as intracellular T-cell epitopes that promote cellular immunity, enabling strong dual humoral and cellular immune activation. The large number of viral proteins displayed at the VLP surface improves the interaction between VLPs and different immune system mechanisms, inducing highly effective, long-lasting cellular and humoral immune responses and leading to optimal production of specific antibodies [90].

BTV is a non-enveloped virus with a triple-layered capsid: the outer layer contains 60 VP2 trimers and 120 VP5 trimers, the intermediate layer comprises 260 VP7 trimers, and the inner shell is formed by 120 VP3 monomers. The outer and intermediate layers are tightly associated, with each VP2 trimer interacting with four VP7 trimers, and each VP5 trimer spanning the channel created by six surrounding VP7 trimers [26].

VLPs against Bluetongue can be generated through the self-assembly of BTV capsid proteins (VP2, VP5, VP3, and VP7) expressed and secreted by bacteria, yeast, or plant expression systems [88,95,96]. Among these capsid proteins, several studies have identified VP2 as the most important component, and its presence is essential in any potential VLP-based vaccine. This phenomenon is illustrated by the fact that a capsid-like particle (CLP) vaccine, which is based only on VP3 and VP7, was constructed and tested but did not elicit protective immunity [89].

Several VLP-based vaccines against BTV have reached different stages of development. For example, a cocktail of VP2, VP3, and VP5 BTV proteins expressed using a baculovirus system yielded promising results, providing protection against five targeted serotypes (BTV-1, BTV-2, BTV-10, BTV-13, and BTV-17). The *Nicotiana benthamiana* transient plant expression system offers an alternative method for VLP production. Plants have become a widely used host for recombinant protein expression, mostly due to their potential to make the system extremely cost-effective [97]. BTV-8 VLPs were successfully produced by transforming *Agrobacterium tumefaciens* with four plasmids encoding capsid proteins, followed by plant infection with the recombinant bacterium. Plant leaves were harvested approximately 10 days later, and the VLPs were purified using extraction, filtration, and several steps of gradient centrifugation. This VLP vaccine elicited protective immunity in vaccinated sheep, which showed neither clinical signs nor viremia following BTV-8 challenge [11,12,97]. Consequently, most bluetongue VLP vaccines, whether developed or currently under development, rely on plant or insect cell expression systems. VLPs developed using these two expression systems have been successfully used as vaccines and have induced strong cellular and humoral immune responses. However, both expression systems have drawbacks, including very high production costs and the possibility of contamination associated with the utilization of insect cells as an expression host. [88,98]. This would probably explain why, despite BTV VLP-based vaccines having demonstrated to be safe and efficacious, no vaccine of this type has reached the commercialization stage to date [11,14]. The main reason for this situation is probably related to the high cost of production; nevertheless, the use of VLPs represents a promising path for the development of safe, efficacious, and DIVA-compatible vaccines [14].

Beyond the economic challenges, bringing VLP-based vaccines to market is often limited by regulatory barriers. Their complex manufacturing processes, the need for consistent production across batches, and strict quality control requirements all complicate approval, slowing large-scale commercialization even when preclinical results are highly promising [91,99].

## 7. Mediterranean Bluetongue Control and Vaccine Strategies: A Critical Discussion

Bluetongue virus remains endemic across the Mediterranean basin, with sporadic epidemic outbreaks driven by *Culicoides* movement, livestock trade, and an expanding climatic niche favorable to the vector. Over the past two decades, multiple serotypes (BTV-1, -2, -4, -8, -9, and -16) have co-circulated continuously in the region [7]. In the early 2000s, Morocco experienced a major outbreak of BTV-4 (230 outbreaks in 2004) and BTV-1 (505 outbreaks in 2006) [100]. This was followed by the rapid spread of BTV-8 across Central and Western Europe and its re-emergence in France in 2015 [42,101]. Other serotypes have re-emerged in the 2010s. BTV-1 reappeared in Corsica in 2013 after a decade of quiescence [39], BTV-4 in Greece and the Balkans, especially in areas with low vaccination coverage, leading to high morbidity and flock-level impact in sheep [102]. The introduction of BTV-3 into Italy (Sicily in 2017 and Sardinia in 2018), likely via wind-borne *Culicoides* from North Africa, proved to be an early signal of a later wave that reached northern Europe [103,104].

From 2019 to 2021, risk assessments documented sustained circulation of this serotype in southeastern Europe and concluded that eradication in vector-rich areas requires prolonged and high vaccination coverage [105]. Additional resurgence events, including BTV-4 in Spain’s Balearic Islands in 2021 [106], and the emergence of a novel BTV-3 variant in the Netherlands in 2023 with rapid spread across neighboring countries by 2024–2025 [107], underscore how quickly new lineages can expand, strain vaccine supply, and trigger movement restrictions [47]. Meanwhile, serotype diversity is expanding beyond the 24 classical serotypes. By 2022, at least 27 serotypes had been confirmed, including the ‘atypical’ BTV-25, -26, and -27, with additional putative BTV-28 to 36 reported from multiple regions. Notably, several atypical serotypes appear capable of vector-independent transmission (limited or no *Culicoides* involvement), enabling silent, year-round circulation [108]. Overall, these recent events point to three durable realities for bluetongue control in the Mediterranean: the sustained multiserotype co-circulation, the continued risk of occasional long-distance viral incursions, and the necessity of integrated control strategies combining rapid diagnostics, movement restrictions, and widespread vaccination where vaccines are available [17,101,102]. From a policy perspective, multiserotype circulation poses a serious challenge. Because protective immunity is largely serotype-specific, effective control depends on either the concurrent deployment of multiple vaccines or the ability to rapidly adapt vaccination strategies as dominant serotypes shift [109]. In practice, delays in vaccine availability following the emergence of new serotypes, such as the recent BTV-3 incursions in Europe, can prolong transmission and amplify economic losses [110,111]. These constraints support the need for regional vaccine banks and harmonized vaccination schedules [17,112]. As the Mediterranean basin effectively functions as a single epidemiologic unit for orbiviruses, control policies must account for cross-border midge movement and livestock trade, coordinating high vaccination coverage in all risk areas to prevent virus refuges in neighboring populations [35,113,114].

Vaccination remains the cornerstone of bluetongue control, and the choice of vaccine platform is critical. Live attenuated vaccines (LAVs) can be multivalent and highly immunogenic, but they have well-known limitations. LAVs may revert or reassort to virulence, lack DIVA capability, and cause viremia in vaccinated animals, posing a transmission risk via *Culicoides* midges. These safety concerns have led many countries to restrict their use [71,77]. Inactivated vaccines are currently the mainstay of bluetongue control in the Mediterranean. They are very safe and effective following booster doses. However, like LAVs, they lack inherent DIVA capability, and repeated administration can be costly and logistically challenging, particularly in large endemic flocks [71]. Next-generation bluetongue vaccines aim to provide broad protection with built-in DIVA capacity. Virus-like particles, which lack a viral genome, elicit strong antibody responses in sheep and, because they omit non-structural proteins, do not induce NSP antibodies, making them inherently DIVA-compatible [71,77]. Recombinant subunit vaccines (purified VP2 or VP2 and VP5 fusions) are likewise safe and DIVA-compatible, although VP2 alone is poorly immunogenic and typically requires potent adjuvants. Viral-vector platforms offer additional design flexibility. For instance, a Modified Vaccinia Ankara (MVA) construct co-expressing a serotype-matched VP2 together with the conserved nonstructural proteins NS1 and NS2 completely protected sheep against homologous challenge and markedly reduced disease after heterologous BTV-8 exposure, while eliciting strong CD8+ T-cell responses [115]. These recombinant vaccines can also be engineered to omit diagnostic antigens (NS3 or VP7), enabling DIVA compatibility. Building on these findings, a dual-track strategy is needed: rapid deployment of safe inactivated vaccines for outbreak control, alongside development of DIVA-compatible, rapidly updateable platforms (VLPs, subunits, and vectored vaccines) for durable, multiserotype protection. This approach should be underpinned by pre-positioned vaccine banks, multispecies coverage, and NSP-based surveillance to maintain detection in vaccinated populations.

Despite the strong experimental promises of next-generation BTV vaccines, there are significant real-world obstacles to their implementation in the Mediterranean region. Novel platforms like VLP and recombinant subunit vaccines have seen limited field implementation due to high production costs and poor scalability [12,71]. Compared with inactivated vaccines, their manufacture is markedly more expensive, especially for bluetongue control and for multivalent formulations. Driven by the need to address the extensive BTV serotype diversity, which requires high antigen yields and stringent quality-control processes, increasing production complexity and reducing economic feasibility [89,116]. Regulatory approval can also be slow for novel platforms; authorities may require extensive safety data, and during that time, countries must rely on existing inactivated vaccines. Deployment is further constrained by logistical limitations, as most veterinary vaccines require strict cold-chain storage (2–8 °C), which is difficult to sustain in hot climates and remote endemic areas, leading to reduced vaccine potency and operational inefficiency [117]. In summary, while next-generation vaccines could greatly enhance bluetongue control (safety, DIVA compatibility, and adaptability), cost, manufacturing scalability, regulatory approval, and delivery logistics remain key barriers. Overcoming these hurdles will be essential before such vaccines can be widely deployed in Mediterranean settings.

Morocco illustrates the transition from sporadic bluetongue introduction to enzootic circulation in Mediterranean settings. Despite long periods without reported outbreaks, the re-emergence of the virus revealed the roles of silent reservoirs and favorable ecological conditions in sustaining transmission [100,118]. Recent surveys show very high BTV prevalence, approximately 42% of sheep and goats tested positive by ELISA between 2018 and 2021, with RT-PCR confirming active BTV infection in roughly 47% of those animals. This high viropositivity demonstrates that BTV is actively circulating within herds, rather than merely reflecting prior exposure. National veterinary records similarly reflect intensive BTV circulation: only 206 cases were reported in 2018, but cases surged to 400–591 in 2019–2020 [100]. These data indicate that Bluetongue is now enzootic in Morocco, with outbreaks recurring whenever environmental conditions favor *Culicoides* midge activity [118].

**Figure 3:**
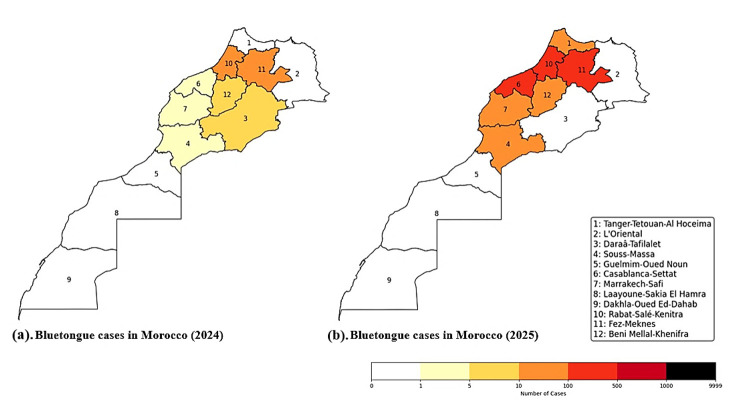
Spatial distribution of reported bluetongue cases in Morocco based on official ONSSA notifications. Choropleth maps display the cumulative number of notified bluetongue cases by administrative region for (**a**) 2024 and (**b**) 2025. Color gradients represent absolute case counts, and numeric labels correspond to the administrative regions listed in the legend. Data were compiled from ONSSA Morocco, covering surveillance reports issued during 2024–2025 [119].

The spatial pattern of reported Bluetongue cases is shown in the choropleth maps (Figure 3) for 2024 and 2025 [119]. In 2024, cases were clustered in the northern and north central regions, notably Tanger-Tetouan-Al Hoceima (1), Rabat-Sale-Kenitra (10), and Fez-Meknes (11), with moderate activity in Beni Mellal-Khenifra (12) and Draa-Tafilalet (3). Southern Saharan regions, Laayoune-Sakia El Hamra (8) and Dakhla-Oued Ed-Dahab (9), show few or no detected cases. By 2025, the intensity and extent of cases increased across the northern half, with Rabat-Sale-Kenitra (10) and Fez-Meknes (11) remaining the major hotspots, and Casablanca-Settat (6) and Marrakech-Safi (7) showing notable rises, while the far south remained comparatively unaffected [119]. Overall, the maps suggest a stable north-center hotspot with a slight south-westward extension along the Atlantic plain patterns consistent with livestock density, animal-movement corridors, and vector-friendly agro-ecological zones. A key feature of Morocco’s Bluetongue situation is the role of different host species in maintaining the virus. The disease affects sheep, goats, and cattle, with sheep being the most clinically susceptible. In contrast, cattle and especially goats often develop silent or subclinical infections, which help sustain BTV transmission. Recent field data show similar BTV seroprevalence in goats and sheep (37.5% vs. 42.6%), with mixed flocks of goats and sheep exhibiting higher infection rates than flocks of sheep alone. According to these results, goats serve as a significant reservoir for BTV, storing the virus without exhibiting any symptoms and enabling its covert spread through nearby *Culicoides* vectors [100].

Historically, Morocco’s vaccination campaigns have focused on sheep, with no systematic bluetongue vaccination program for goats [100]. This is a critical gap, as unvaccinated goat populations can act as a continual source of infection, reseeding the virus. This case study underscores the importance of a multi-species approach to Bluetongue control in endemic regions. Indeed, Bluetongue often spreads “quietly” between recognized outbreaks because surveillance hinges on clinical cases in sheep. Tolerance in local breeds and immunity from prior exposure can mask new transmission until susceptible young stock or a more virulent strain emerges and causes obvious disease. With multiple serotypes, such as BTV-1 and BTV-4, co-circulating, reassortment remains a risk. While Morocco has avoided emergent reassortants causing major outbreaks, the risk persists, underscoring the need for vigilant monitoring, including expanded vaccination coverage and vector control [17,101,102].

Current bluetongue surveillance in the Mediterranean still has significant blind spots. Although BTV infections have been detected in wild ruminants following major outbreaks [120], routine surveillance remains largely focused on livestock, with wildlife and unmanaged animal populations rarely included [121]. This gap creates a risk that the virus may overwinter or persist silently in wildlife reservoirs, delaying outbreak detection and undermining control efforts.

At the same time, BT control is still mostly managed as a national issue, despite the clearly transboundary nature of the disease. Infected *Culicoides* midges and animal movements allow outbreaks in one country to quickly threaten neighboring regions [109,122], and long-distance wind dispersal of vectors has been confirmed as a major introduction pathway, as illustrated by the spread of BTV-3 from Sardinia [122].

Across the Mediterranean, these challenges are further compounded by the concurrent circulation of other transboundary animal diseases, such as peste des petits ruminants (PPR), which also require large-scale vaccination and movement controls [123,124]. Managing multiple, disease-specific vaccination campaigns puts pressure on veterinary resources and risks reducing farmer engagement, highlighting the need for a more coordinated approach. In this context, a broader One Health or episystem framework, integrating surveillance, data sharing, and synchronizing vaccination campaigns across diseases and borders, appears efficient and essential to achieve more resilient and sustainable bluetongue control [109].

## 8. Future Directions in Bluetongue Vaccine Development

Vaccination has proven effective in controlling Bluetongue outbreaks, as evidenced by past epidemic control efforts. However, while most available vaccines are effective, they have significant limitations that are becoming more apparent as the demand for vaccination increases [12,13]. Although live attenuated vaccines are effective and cost-effective, they are not completely safe because of the potential reversion of the virus to virulence and the possibility of reassortment events with virulent serotypes that could lead to the emergence of new virulent strains. On the other hand, inactivated bluetongue vaccines eliminate these risks but remain costly and typically require multiple booster doses [125]. Crucially, neither inactivated nor live attenuated vaccines can differentiate between infected and vaccinated animals (DIVA), and the protection they offer is limited to the serotype contained in the vaccine. All these factors underline the need for next-generation BTV vaccines that move beyond serotype-specific, non-DIVA [13].

The immune response that must be induced to confer protection is well known, and VP2, alone or in combination with other BTV proteins (VP5), can induce this response and can therefore be used to develop subunit vaccines for disease control, with the added advantage of being DIVA compatible. Similarly, VLP-based BTV vaccines also represent interesting prospects; they have been shown to be even more effective than subunit vaccines and can also be made DIVA-compatible by the introduction of specific markers, allowing safer animal movement without the risk of BTV disease spread [69]. Recombinant vaccine technologies are emerging as the predominant strategy for the next generation of bluetongue virus immunization. The flexibility of molecular methods would allow adjuvants to be added and bluetongue vaccines to be developed as vectors for other subunit vaccines or capable of providing immunity against several different serotypes [125,126].

In this context, multiepitope vaccines may represent a particularly promising alternative: by combining immunodominant epitopes from multiple BTV proteins, these vaccines can induce broad, cross-protective immunity across several serotypes, while remaining safe, DIVA-compatible, and amenable to cost-effective production [126].

## 9. Conclusions

**Figure 4:**
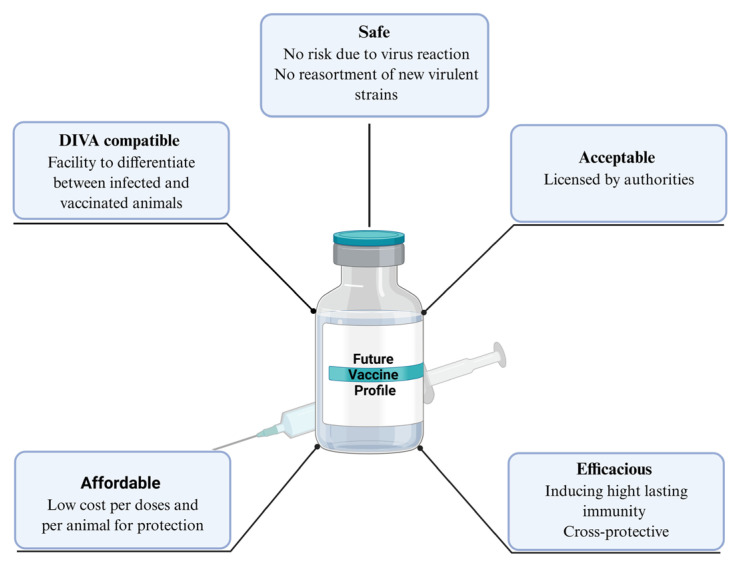
Conceptual summary illustrating the characteristics of future vaccines against BT. The most important characteristics to consider in the design and development of new bluetongue vaccines are safety, efficacy, and cross-protection, licensing, DIVA compatibility, and affordability. Created in BioRender. Joubair, I. https://BioRender.com/fxntmwg (accessed on 9 April 2025).

In summary, in the Mediterranean Basin, where bluetongue epidemiology is defined by serotype diversity, silent reservoirs, and recurrent incursions driven by climate and animal movement, the development of next-generation vaccines must address these region-specific complexities. An ideal next-generation vaccine would confer rapid, durable immunity across multiple serotypes and ruminant species, remain safe and cost-effective, and permit DIVA-compliant surveillance (Figure 4) [13]. Achieving this will require leveraging molecular design and rigorous evaluation of cross-protective efficacy in relevant animal models.

## Figures and Tables

**Table 1:** Comparison of the VP2, VP5, VP3, and VP7 nucleotide sequences of serotypes 1, 4, and 8.

Compared Sequences		Results	
		Identity	Similarity
VP2	S1(MT070930.1)/S4(PV926265.1)	56.3%	56.6%
	S1(MT070930.1)/S8(PV974536.1)	59.1%	59.4%
	S4(PV926265.1)/S8(PV974536.1)	73.91%	-
VP5	S1(MT070934.1)/S4(PV926269.1)	68.6%	69.2%
	S1(MT070934.1)/S8(PV974530.1)	72.7%	73.4%
	S4(PV926269.1)/S8(PV974530.1)	70.43%	-
VP7	S1(MT070935.1)/S4(PV926270.1)	94.2%	95.1%
	S1(MT070935.1)/S8(PV974541.1)	91.9%	92.8%
	S4(PV926270.1)/S8(PV974541.1)	96.71%	-
VP3	S1(KP696574.1)/S4(PV926266.1)	96.5%	96.9%
	S1(KP696574.1)/S8(PV974537.1)	93.5%	93.9%
	S4(PV926266.1)/S8(PV974537.1)	96.18%	-

## Data Availability

No new data were created or analyzed in this study. Data sharing is not applicable to this article.
